# MRP Transporters and *Low Phytic Acid* Mutants in Major Crops: Main Pleiotropic Effects and Future Perspectives

**DOI:** 10.3389/fpls.2020.01301

**Published:** 2020-08-27

**Authors:** Federico Colombo, Dario Paolo, Eleonora Cominelli, Francesca Sparvoli, Erik Nielsen, Roberto Pilu

**Affiliations:** ^1^Department of Agricultural and Environmental Sciences—Production Landscape, Agroenergy, Università degli Studi di Milano, Milan, Italy; ^2^Institute of Agricultural Biology and Biotechnology, Consiglio Nazionale delle Ricerche (CNR), Milan, Italy; ^3^Department of Biology and Biotechnology, Università degli Studi di Pavia, Pavia, Italy

**Keywords:** low phytic acid mutants, phytic acid, multidrug resistance-associated-protein transporters, pleiotropic effects, nutritional and environmental problems

## Abstract

Phytic acid (PA) represents the major storage form of seed phosphate (P). During seed maturation, it accumulates as phytate salts chelating various mineral cations, therefore reducing their bioavailability. During germination, phytase dephosphorylates PA releasing both P and cations which in turn can be used for the nutrition of the growing seedling. Animals do not possess phytase, thus monogastric animals assimilate only 10% of the phytate ingested with feed, whilst 90% is excreted and may contribute to cause P pollution of the environment. To overcome this double problem, nutritional and environmental, in the last four decades, many low phytic acid (*lpa*) mutants (most of which affect the PA-MRP transporters) have been isolated and characterized in all major crops, showing that the *lpa* trait can increase the nutritional quality of foods and feeds and improve P management in agriculture. Nevertheless, these mutations are frequently accompanied by negative pleiotropic effects leading to agronomic defects which may affect either seed viability and germination or plant development or in some cases even increase the resistance to cooking, thus limiting the interest of breeders. Therefore, although some significant results have been reached, the isolation of *lpa* mutants improved for their nutritional quality and with a good field performance remains a goal so far not fully achieved for many crops. Here, we will summarize the main pleiotropic effects that have been reported to date in *lpa* mutants affected in PA-MRP transporters in five productive agronomic species, as well as addressing some of the possible challenges to overcome these hurdles and improve the breeding efforts for *lpa* mutants.

## Introduction

**Figure 1:**
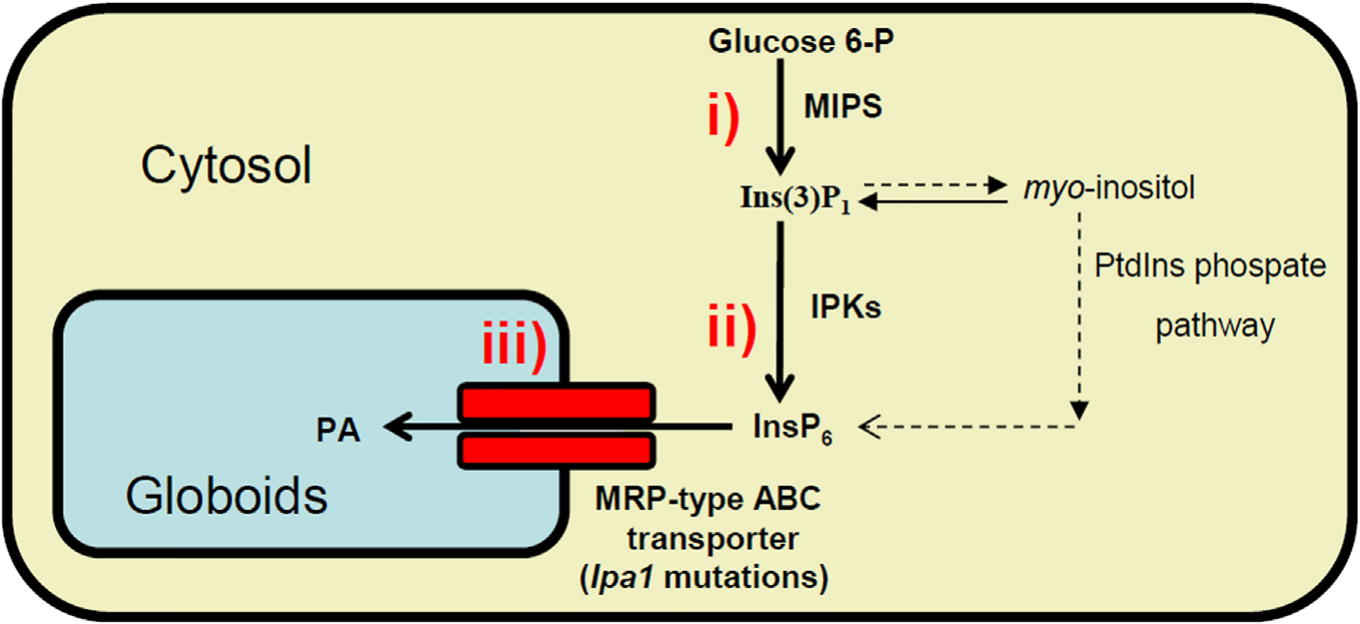
Schematic view of the biosynthetic pathways leading to PA accumulation in globoids (storage vacuoles) in seeds. *lpa* mutations can be divided into three classes: i) mutations affecting the activity of *myo*-inositol 3-phosphate synthase (MIPS); ii) the successive phosphorylation steps of PA pathway, from Ins(3)P1 to the accumulation of PA; iii) transport by MRP transporters and storage of PA into the globoids.

In plants, phytic acid (PA) (*myo*-inositol-1,2,3,4,5,6-hexa*kis*phosphate) represents the major storage form of phosphate (P) in seeds (up to 85%) (Raboy, 1997). PA is synthesized in the endoplasmic reticulum and during maturation it is deposited in the protein storage vacuole inside inclusions named globoids (Raboy, 2002). The location of the PA reserve inside the seed varies depending on the species: in maize it is mainly accumulated in the embryo and in the scutellum, while in rice and wheat, 80% of PA is found in aleurone and maternal teguments, and only small quantities are in the embryo (O’Dell et al., 1972). In legumes, such as soybean and common bean, more than 95% of PA is found in cotyledons (Ariza-Nieto et al., 2007). Due to its high negative charge at the physiological pH, PA chelates cations (such as iron, zinc, potassium, calcium, magnesium), forming poorly bioavailable phytate salts. During germination, phytase and other enzymes degrade PA releasing *myo*-inositol, orthophosphate and cations, which can be remobilized to support seedling growth (Laboure et al., 1993). Among animals feeding on seeds, only ruminants can degrade PA thanks to the presence in their digestive systems of bacteria endowed with phytase activity. However, monogastric animals (including humans) possess almost no phytase activity in the digestive tract, thus they degrade only about ~10% of the phytate in the feed, while ~90% is excreted. Therefore, farmers breeding pigs, poultry, fish, and other monogastric animals must provide supplementary feed with mineral phosphorus and cations. Due to the paucity of the global inorganic P reserves, this in turn implies an economic problem. Moreover, the excreted amount of PA-derived P is high in manure, and consequently in soils, thus contributing to P pollution and to eutrophication of groundwater, a serious environmental problem (Raboy, 2009). For these reasons, PA is considered an anti-nutritional compound and its reduction or elimination in cereal and legume seeds has been and is still an important challenge in plant breeding programs. Among the different strategies used to achieve this result, many low phytic acid (*lpa*) mutants have been isolated in all major crops (Naidoo et al., 2012; Sureshkumar et al., 2014). These mutants may have some advantages, mainly (i) improving phosphorus management in non-ruminant production, (ii) contributing to enhance sustainability and reduce animal waste P, and (iii) increasing mineral bioavailability as a strategy to combat mineral deficiencies, as recently reviewed (Raboy, 2020). According to the step of the PA biosynthetic pathway, *lpa* mutations can be divided into three categories: 1) mutations affecting the first step in which *myo*-inositol 3-phosphate synthase (MIPS), the first enzyme of the biosynthetic pathway, transforms glucose 6-P into *myo*-inositol(3)-monophosphate leading to a relevant decrease in PA accumulation and a simultaneous increase in inorganic phosphate (Pi); 2) mutations in different genes coding for enzymes involved in the successive phosphorylation steps of PA pathway, from *myo*-inositol(3)-monophosphate to PA leading to accumulation of inositol phosphates (InsPs) intermediates which represent a distinctive characteristic only for this second class of mutants; 3) mutations affecting the transport and storage of PA into the vacuole through the multidrug resistance-associated-protein (MRP) transporters (**Figure 1**). In the last category of mutants, PA is exposed to the attack of dephosphorylating enzymes, thus strongly decreasing the amount of PA and increasing that of Pi, the same features registered also in the first category of mutants. This similarity between categories 1 and 3 generated a lot of confusion in the first characterization of some mutations in PA transporter genes. In fact, the first experiments carried out to map the maize *lpa1* mutation seemed to reveal a lesion in a member of the gene (located on chromosome 1, coding for *MIPS*). Moreover, in mutants affecting *ZmMRP4*, *ZmMIPS1S* expression is reduced (Raboy et al., 2000; Pilu et al., 2003; Shukla et al., 2004; Shi et al., 2007); later, mapping and expression data found that in maize both *ZmMIPS* and *ZmMRP4* map very closely on chromosome 1S. A few years later, transposon mutagenesis experiments performed by Shi et al. demonstrated that *ZmMRP4* (accession number EF86878), coding an MRP, is the gene responsible for *lpa1* mutation (Shi et al., 2007). All these mutations cause the lack of PA transfer from the cytosol into the storage location of the vacuole. This, in turn, probably exposes PA to a dephosphorylation process carried out by cytosolic phosphatases, thus remarkably decreasing the final amount of phytate and simultaneously increasing that of free Pi and cations, which during maturation accumulate into vacuolar protein bodies in seed storage tissue.

A high proportion of these *lpa* mutants have been shown to carry mutations in genes coding for MRPs. These proteins belong to the ABCC cluster of plant ATP-binding cassette (ABC) transporters found in many species which translocate anions of various organic molecules across intra-cellular membranes (Shi et al., 2007; Gillman et al., 2009; Nagy et al., 2009; Xu et al., 2009; Panzeri et al., 2011; Sparvoli and Cominelli, 2014; Cominelli et al., 2020b). Such a class of mutants appears the most interesting one, since it shows the highest drop in PA level together with a concomitant substantial increase of free P and a consequent supposed increase in free cations. Unfortunately, almost all *lpa* mutations described during the last four decades, including the ones affecting the PA-MRP transporters, are associated with poor agronomic performance which is linked to many negative pleiotropic effects regarding mainly (but not exclusively) seed viability and plant development (Raboy et al., 2000; Meis et al., 2003; Guttieri et al., 2004; Pilu et al., 2005; Bregitzer and Raboy, 2006). Pleiotropic effects in *lpa* mutants may be ascribed to the pivotal role of inositol metabolites as signaling molecules in key cellular pathways, such as hormonal perception, epigenetic control of the chromatin landscape, cellular trafficking and calcium homeostasis (Sparvoli and Cominelli, 2015). In plants it is almost accepted that InsP_6_ instead of InsP_3_ is involved in signaling. The first evidence was the finding that, in guard cells, InsP_6_ triggers intracellular Ca^2+^ release after ABA addition with an efficiency ≈100 times higher than that of InsP_3_ (Lemtiri-Chlieh et al., 2003). However, despite evidences for the signaling pathway, canonical InsP_3_/InsP_6_ receptors have never been reported in plants. These mutants have received very little interest until now, mainly due to their negative pleiotropic effects. However, a recent analysis suggested that the choice of strategies alternative to the use of *lpa* mutants (such as the addition to animal feed of P or phytase to increase the component of available phosphorus) has been done without calculating the possible long-term money-saving deriving from using the *lpa* crops (Raboy, 2020).

The present review focuses in particular on the pleiotropic effects reported to date in cereals’ and legumes’ *lpa* mutants affected in PA-MRP transporters, which have disclosed a number of very interesting clues to shed more light on seed physiology and to offer tools suitable to develop biotechnological and sustainable approaches aimed at improving food and feed.

## MRP-Type ABC Transporters and PA Transport

**Figure 2:**
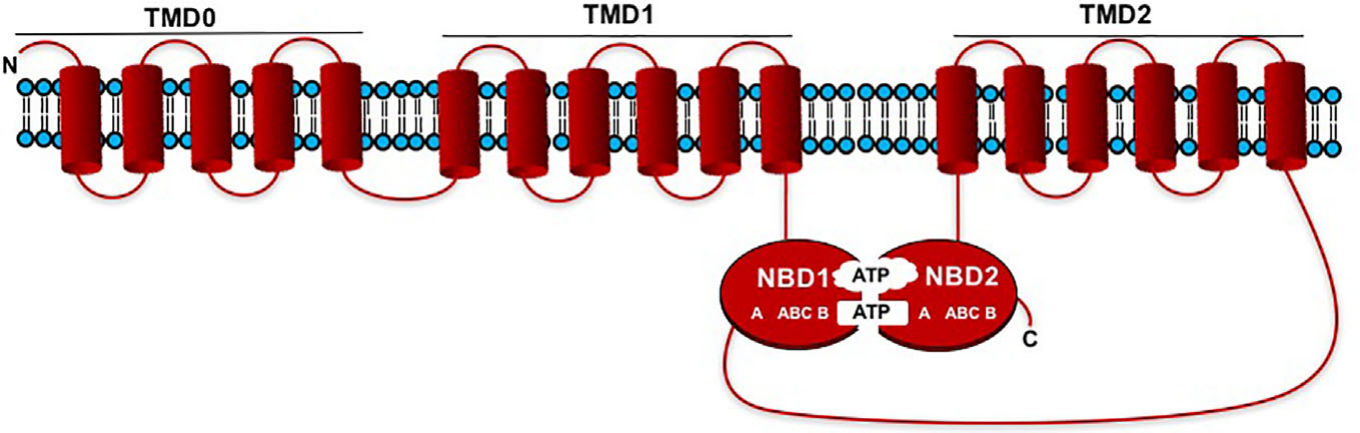
Schematic representation showing the membrane topology models of MRP-type ABCC transporters. The domains are forward oriented in the following way: TMD0_TMD1-NBD1_TMD2-NDB2. NBDs contain the Walker A and Walker B motifs separated by an ABC “signature”.

ABC transporters are plant transmembrane transporters that beside being involved in the transport of molecules necessary for plant growth (hormones, lipids, metabolites, and defense compounds) across cell membranes, are involved in different plant processes, such as xenobiotic detoxification, regulation of stomatal guard cell movements, and oxidative stress tolerance (Gaedeke et al., 2001; Swarbreck et al., 2003; Klein et al., 2006; Hwang et al., 2016). In most cases, the driven transport occurs against electrochemical gradients using the energy supplied by ATP hydrolysis (Wilkens, 2015). ABC transporters are ancient macromolecules widespread in all organisms, and in plants 8 subfamilies have been identified. They are generally characterized by a common structure composed of two soluble nucleotide-binding domains (NBD1, NBD2) and two hydrophobic transmembrane domains (TMD1, TMD2), which contain six transmembrane α-helices (**Figure 2**). NBDs contain the Walker A and Walker B motifs separated by around 120 amino acids as well as an ABC “signature”. In most cases domains are forward-oriented in the following way: TMD1-NBD1_TMD2-NBD2, however the NBDs and TMDs may be arranged in the opposite fashion: NBD1-TMD1_NBD2-TMD2, and ABC transporters “made up by half-size” units also exist (Verrier et al., 2008). MRP proteins belong to the ABCC cluster of plant ABC transporters. Unlike other ABC transporters, MRP proteins are characterized by an additional extremely hydrophobic N-terminal extension (TMD0) consisting of around 220 amino acids. TMD0 contains five transmembrane α-helices, it is positioned before TMD1 and is connected *via* a cytosolic loop (CL3) to the rest of the protein (Sparvoli and Cominelli, 2014) (**Figure 2**). These proteins share a very high degree of similarity among different species (Cominelli et al., 2020a). The role of TMD0 in plants is not yet defined, while normally the CL3 portion plays a key function in the recognition and transport of the substrate (Gao et al., 1998). In 2007 Shi et al. isolated the maize *lpa1* mutation affecting the *ZmMRP4* gene (accession number EF586878), through the screening of a transposon mutagenized plant collection. These authors demonstrated for the first time that an MRP-type ABC transporter was required for PA transport (Shi et al., 2007). This finding was biochemically confirmed in 2009 by Nagy and co-workers who isolated a mutant in the *Arabidopsis thaliana AtMRP5* gene, ortholog of *ZmMRP4* (Nagy et al., 2009). This gene had been characterized a few years earlier for functions apparently unlinked to PA transport, such as root growth, lateral root formation, stomatal movement regulation, anion transport, water use efficiency and guard cell hormonal signalling (Gaedeke et al., 2001; Klein et al., 2003; Suh et al., 2007). As a result of these findings, other *PA-MRP* genes and their corresponding mutants were later characterized in species of agronomic interest such as *Oryza sativa* L. (Liu et al., 2007; Xu et al., 2009), *Glycine max* (L.) Merr. (Gillman et al., 2009; Saghai Maroof et al., 2009; Gillman et al., 2013), *Phaseolus vulgaris* L. (Panzeri et al., 2011; Cominelli et al., 2018), *Triticum aestivum* (RNAi lines in the *ABCC13* genes), (Bhati et al., 2016) (**Supplementary Material Table 1**). Although the gene structure (exon-intron arrangement) of PA-MRP transporters is similar in the different crops (Cominelli et al., 2020b), the main difference between cereals (excluding the hexaploid wheat harbouring three different *ABCC13* genes) and legumes is in gene number: maize and rice are characterized by a single gene copy (*ZmMRP4* and *OsMRP5*, respectively), while legumes have two or three paralogues: *PvMRP1* and *PvMRP2* in common bean and *GmMRP3*, *GmMRP13*, and *GmMRP19* in soybean (Panzeri et al., 2011; Sparvoli and Cominelli, 2014; Cominelli et al., 2018). Indeed, these two species shared a whole-genome duplication event (Lavin et al., 2005) and later soybean underwent another independent whole-genome duplication (Schmutz et al., 2010). PA-MRP protein sequences are highly conserved, even if it is not well known which amino acid residues are involved in PA transport. A multiple alignment of the amino acid sequences in comparison with the sequence of Arabidopsis ABCCs, highlighted some peculiarities: a conserved stretch of lysine residue (found in the cytosolic loop between NBD1 and TMD2), but also the fact that several amino acid residues (Lys and Arg) located in the two TMD domains, seem to be involved in PA transport (Sparvoli and Cominelli, 2014).

## Pleiotropic Effects of *lpa* Mutations in *PA-MRP* Genes

The use of *lpa* mutations, in terms of increasing nutritionally cation bioavailability in the diet, enhancing phosphorus management and reducing environmental impact due to reduced P waste in non-ruminant production, could be an important tool to increase the sustainability of agricultural production.

Unfortunately, *lpa* mutations, including the ones affecting the *PA-MRP* genes, are frequently accompanied by negative pleiotropic effects visible either at the level of seed or plant, thus limiting the interest of breeders (Raboy et al., 2000; Meis et al., 2003; Pilu et al., 2005; Landoni et al., 2013; Raboy et al., 2020).

To study the pleiotropic effects of mutations in the PA-MRP transporters, it is important to take also into consideration the possible variation in the content of inositol pyrophosphates (PP-InsP), caused by the mutation. A small pool of PA present in the cell is further phosphorylated to form PP-InsP, containing one or two diphosphate groups (InsP_7_ and InsP_8_, respectively). PP-InsP have important roles in energy metabolism, hormone signaling (mainly jasmonate), and Pi sensing (Freed et al., 2020). A recent review pointed out that different Arabidopsis *lpa* mutations affecting PA biosynthetic genes, also cause a reduction in the content of InsP_8_ and in some cases of InsP_7_. Due to the important role of these molecules, a decrease in their content may affect pathogen response and Pi sensing (Freed et al., 2020). On the other hand, the Arabidopsis *mrp5* and the maize *lpa1* mutants show increased content of both InsP_7_ and InsP_8_. Hence, from this point of view, *PA-MRP* genes can be considered an interesting target for the development of *lpa* mutants not compromised in P homeostasis and in jasmonate signaling (Freed et al., 2020).

**Figure 3:**
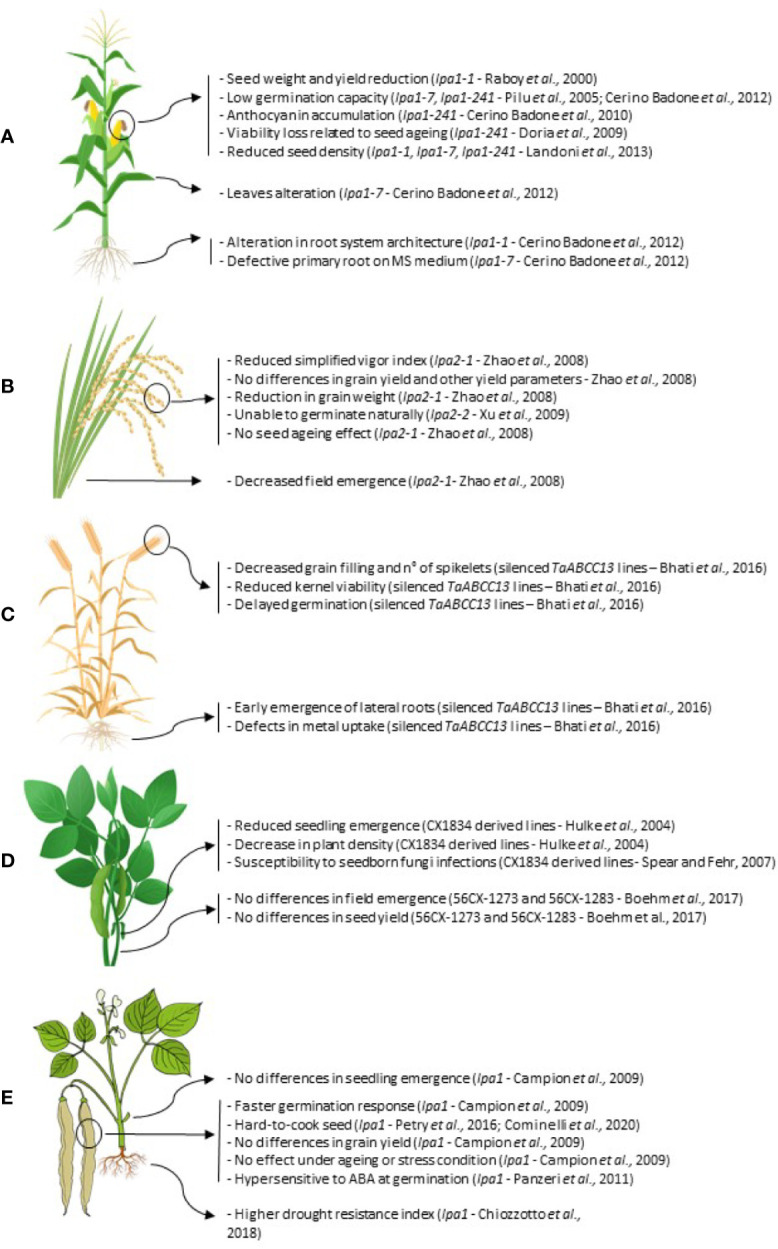
Summary of the main pleiotropic effects in *lpa* mutations in the five agronomic species considered: maize **(A)**; rice **(B)**; hexaploid wheat **(C)**; soybean **(D)** and common bean **(E)**.

In the following sections, we will describe the main pleiotropic effects so far reported in *lpa* mutants affected in PA-MRP transporters in five important productive agronomic species: maize, rice, wheat, soybean, and common bean (**Figure 3**).

### Maize

In maize, *lpa1* mutations are caused by lesions in the *ZmMRP4* gene. Four important mutations have been isolated so far in the *ZmMRP4* PA transporter: *lpa1-1*, consisting of a point mutation that determines an A1432V substitution (Shi et al., 2007); *lpa1-241*, a paramutagenic allele described by Pilu et al. which causes a series of negative pleiotropic effects depending on its strength (Pilu et al., 2005; Pilu et al., 2009); *lpa1-7*, probably determined by a mutation in the coding sequence, even if the nature of a paramutagenic allele can be discarded due to its stability (Cerino Badone et al., 2012); *lpa1-5525*, a recently found *lpa1* mutant allele obtained by transposon tagging mutagenesis (Borlini et al., 2019), but not yet fully characterized. All these mutations lead to a reduction in the kernel PA content, accompanied by a proportional increase in P_i_, even if the total P remains unchanged. In particular, *lpa1-1* allele shows a 66% reduction in PA content and is viable in its homozygous state (Raboy et al., 2000), while in the case of *lpa1-241* and *lpa1-7* mutants, displaying a drop in PA content greater than 80%, germination is suppressed (Pilu et al., 2005; Cerino Badone et al., 2012). Among the negative pleiotropic effects associated with these maize mutants, a seed weight reduction ranging from 8 to 23% characterized *lpa1-1* (Raboy et al., 2000). This decrease appears to be mainly caused by endosperm loss and consequently results in an agronomic yield reduction. Concerning this mutant, it was also observed that under field conditions, *lpa1-1* is more susceptible to drought stress, probably due to an alteration in mature root system development (Cerino Badone et al., 2012).

The *lpa1-241* mutant showed a variety of morphological and physiological changes of which the negative effects appear connected to the “strength” of the mutation. In fact, in the *lpa1-241* mutants the PA content is variable and it was shown that individual seeds with less than 20% of wild type PA content are unable to germinate (Pilu et al., 2005). Such an observation might be explained by the finding that an imperfect alignment between root and shoot primordia occurs, thus introducing an asymmetry in the body plan (Pilu et al., 2005; Pilu et al., 2009). An embryo-rescue technique (embryos removed from the seed and transferred to Murashige and Skoog, MS medium) allowed the restoration of high germination capacity in *lpa1-241* seeds, even if many defective seedlings were found and their growth was slower compared to wild type.

The characterization studies carried out on the same maize *lpa1-241* mutant allowed the discovery of a hitherto unknown role of the PA presence in the seed. In fact, Doria and co-workers used this mutant as a tool to study the consequences of the lack of this important reserve substance on seed survival and longevity (Doria et al., 2009). In this study the focus was on iron homeostasis; in the anaerobic cell environment the oxidation of unchelated Fe^2+^ to Fe^3+^ is a potential source of Reactive Oxygen Species, considered the main cause of the viability loss related to seed ageing.

Due its ability to remove cations, PA was hypothesized to be a good candidate for protecting the embryo from such oxidative processes. Consequently, these authors collected data on germinability, free iron level, free radical relative abundance both by EMR (Electronic Magnetic Resonance) and histological evidence, protein carbonylation level, amount of damage to DNA, degree of lipid peroxidation, tocopherol level and antioxidant capacity level of seeds of maize B73 (control) and of an isogenic low phytic acid mutant (*lpa1-241*), either unaged or incubated for 7 days in accelerated ageing conditions (46 C° and 100% relative humidity). Results clearly demonstrated that *lpa1-241* mutant seeds, compared to the wild type ones, show: 1) a lower germination capacity, which decreased further after accelerated ageing; 2) about 50% more free or weakly bound iron; 3) upon accelerated ageing, an higher content of free radicals mainly concentrated in the embryo, a higher extent of carbonylation of seed proteins and of damage (apurinic/apyrimidinic sites) on DNA, whereas lipids did not appear more peroxidated, although γ-tocopherol content was decreased by about 50%, probably because it is consumed just to prevent membrane peroxidation; 4) an higher level of antioxidants such as total glutathione and tocopherol, the synthesis of which is probably induced by the increase of ROS, as well as a higher level of anti-radical power measurable by the DPPH test.

These findings were interpreted in terms of previously reported, but never proven, antioxidant activity of PA through iron complexation. In conclusion, behind the fundamental role of P and cations storage, PA appears to play another important function consisting in the protection of embryo viability from oxidative stress during seed maturation and dormancy.

Another pleiotropic effect in *lpa1-241* concerns the accumulation of anthocyanins in the kernel.

In fact, Goodman *et al*. observed that *ZmMRP4* is expressed in the aleurone layer and is co-regulated with another MRP protein (*ZmMRP3*), expressed in all tissues accumulating anthocyanins, particularly in the husk suggesting that it is somehow involved in anthocyanin transport (Goodman et al., 2004).

These expression data were confirmed in lines carrying both the *lpa1-241* mutation and the alleles of the genes involved in anthocyanin biosynthesis active in the kernel, a change from red to bluish color occurred in the scutellum of the *lpa1-241* mutant kernel, thus suggesting a possible role of *ZmMRP4* in the transport of this pigment: in fact, when anthocyanins are transported in the vacuole, due to the acid pH, they assume the typical reddish color, but if MRP is not functional, they are not transported and accumulate in the less acid environment of the cytosol where they retain the bluish tint (Badone et al., 2010).

As well as the *lpa1-241* mutant, also *lpa1-7* showed several agronomic defects due to the strong reduction (>80%) in PA content (Cerino Badone et al., 2012). Compatibly with a recessive monogenic behavior, an inability to germinate was observed in both filter paper germination tests and in field conditions. This mutation was lethal in the homozygous state (*lpa1-7*/*lpa1-7*), although the embryo rescue technique could recover the germination capacity. Among other pleiotropic effects, seedlings on MS medium were characterized by slow growth and defective primary roots, partially compensated by the development of secondary roots. Moreover, the leaves of homozygous *lpa1-7* plants showed alterations compared to the wild type and light green stripes between leaves’ venation were clearly visible (Cerino Badone et al., 2012). These observations were confirmed by data showing a decrease in chlorophyll, carotenoid and trichome length as well as an increase in trichome density (Cerino Badone et al., 2012). Histological analysis aimed at comparing features of *lpa1-7*/*lpa1-7* and wild type kernels highlighted a reduction in the mutant embryo dimension and a misalignment between the radical primordium and the embryo body in *lpa1-7* (Cerino Badone et al., 2012).

A different seed density between all *lpa1* mutants and the respective wild types was highlighted (Landoni et al., 2013). In the same work, the *lpa1-7* mature kernel was characterized by a clearly visible cavity in the endosperm, that was absent in the wild type.

### Rice

The rice ortholog of *ZmMRP4* is *OsMRP5*; the two proteins share 83% of nucleotide sequence identity and 91% of amino acid identity (Shi et al., 2007). Different mutants at the *OsMRP5* locus have been identified (Liu et al., 2007; Xu et al., 2009): *Os-XS-lpa2-1* consisting of a point mutation in the sixth exon that causes a P1156S substitution in TMD2 (Xu et al., 2009); *Os-XS-lpa2-2*, a 5 bp deletion in the first exon that leads to a frame shift at amino acid 452, causing the occurrence of a premature stop codon at the amino acid 474 (Xu et al., 2009). *Os-XS-lpa2-1* is characterized by a 20% reduction in PA content and is not lethal when in homozygosity. However, the PA decrease was found to be much higher in the lethal mutant *Os-XS-lpa2-2* (>90%). Moreover, a T-DNA knock out line (4A- 02500) in which *OsMRP5* was disrupted, showed the same reduction in PA content (~90%) and appeared lethal in the homozygous state (Xu et al., 2009). Comparing *Os-XS-lpa2-1* mutants with their respective wild types, different pleiotropic effects associated with reduced seed viability and plant performance were pointed out (Zhao et al., 2008). The simplified relative vigor index (a parameter that combines germination rates, seedling height and seedling weight) is reduced by 7.8% in *Os-XS-lpa2-1*, despite a germination rate which is similar to that of wild type. Moreover, a significant decrease in field emergence rate was observed (65% in *Os-XS-lpa2-1*, versus 84% in the wild type), while a 5% reduction in grain weight was measured in the mutant. Conversely, no significant differences were found in grain yield and other yield parameters, such as ripened grain rate, number of grains per panicle and number of productive tillers (Zhao et al., 2008). In the same work, an artificial ageing test was performed on mutant rice seeds (42°C and 95% relative humidity for 7 and 14 days), but in contrast to what was observed in the maize *lpa 241-1* mutant described previously, no significant difference was found. This discrepancy might be attributed to the different location of the PA deposits, 90% of which in rice seed are in the aleurone tissue, whilst in maize seed they are in the germ, which is obviously a much more critical location in relation to the maintenance of the germination ability. So, according to this theory, rice seed might endure much better than maize the oxidative stress connected with the paucity of PA.

*Os-XS-lpa2-2* is an allelic mutant of *Os-XS-lpa2-1* and is characterized by severe agronomic defects. Due to the strong reduction in PA (>90%), this mutant cannot germinate naturally, but seedlings can be produced from immature embryos through *in vitro* culture on MS medium (Xu et al., 2009).

### Wheat

In hexaploid wheat, three copies of the *TaABCC13* gene are present and the encoded proteins show a high degree of similarity with the other cereal PA-MRP transporters (Cominelli et al., 2020b). The TaABCC13 proteins have been previously described as cadmium transporters (Bhati et al., 2015). In a subsequent publication, the *TaABCC13* genes were silenced through RNA-interference (RNAi) and in the silenced lines, a reduction of 22–34% in seed PA content was observed. Moreover, these lines were characterized by a decrease in grain filling, numbers of spikelets, kernel viability, delayed germination, early emergence of lateral roots, and defects in metal uptake and development of lateral roots in the presence of cadmium stress, compared to the non-transgenic lines. These data show that *TaABCC13* is important for several other aspects of growth, as well as for grain nutritional quality, for root development and detoxification of heavy metals (Bhati et al., 2016).

A common alteration in the maize *lpa1* mutant and in the silenced *TaABCC13* wheat lines refers to defects in root growth and development (Cerino Badone et al., 2012; Bhati et al., 2016). In a previous work, it was shown that the Arabidopsis *mrp5-1* mutant seedlings, grown on standard medium (0.5 x Murashige and Skoog -MS- medium), showed a reduction in primary root elongation, accompanied by an earlier growth of lateral and secondary roots. However, when seedlings were grown on a more complete medium (1x MS medium), a reverse phenotype was obtained. A two-fold increase in auxin content was also recorded in roots of *mrp5-1* seedlings compared to the wild type ones when grown on standard medium (Gaedeke et al., 2001), indicating that PA transport is important for auxin accumulation and signaling. The phenotypic alterations in root growth and development described in crops are similar to the ones described in Arabidopsis that can be considered as a model system to further study these aspects.

### Soybean

Mutants in PA-MRP transporters were found not only in cereals, but also in legumes (Wilcox et al., 2000; Campion et al., 2009; Cominelli et al., 2018). In soybean, chemical mutagenesis was used on the breeding line *CX1515-4* and two independent and non-lethal *lpa* mutants were isolated: *M153* and *M766* (Wilcox et al., 2000). Although the initial analysis of the *M153* line suggested that only a single locus was responsible for the *lpa* phenotype, a few years later it was found that the low phytate trait was controlled by two recessive alleles at two independent loci, initially called *pha1* and *pha2* and subsequently renamed *lpa1* and *lpa2* (Oltmans et al., 2004; Gao et al., 2008). These loci correspond to two genes (*Gm03g32500* and *Gm19g35230*) that code for PA-MPR transporters (GmMRP3 and GmMRP19) which are mutated in the independent soybean line *CX1834* deriving from *M153* (Gillman et al., 2009; Saghai Maroof et al., 2009). It was shown that these transporters are homologous to *ZmMRP4* and *AtMRP5* (Gillman et al., 2009; Saghai Maroof et al., 2009; Gillman et al., 2013). A third MRP protein, GmMRP13 (*Gm13g18960*), was identified on chromosome 13 (Panzeri et al., 2011). In the *M153* line, the *lpa1-a* allele carries a nonsense mutation at R893, which results in a truncated protein (Gillman et al., 2009; Saghai Maroof et al., 2009), while the *lpa2-a* allele causes a R1039K change. In *M766*, the *lpa1-b* allele is characterized by a T>A SNP at intron 9, which introduces an alternative splicing site; the *lpa2-b* allele shows a single base change in position 1039 (as in *lpa2-a* allele) that results in an early termination (Gillman et al., 2013).

Both *M153* and *M766* are characterized by a significant decrease in PA content (80 and 76.3% respectively), although the greatest drop in PA (94% less compared to the parental line) was achieved in the double mutant, obtained by combining the *lpa1-a* allele from *M153* and the *lpa2-b* allele from *M766* (Wilcox et al., 2000; Oltmans et al., 2004; Oltmans et al., 2005; Gillman et al., 2013). As in cereals, this strong PA reduction is often associated with negative pleiotropic effects. The first agronomic trials were conducted with lines derived from *M153*. Comparing these *lpa* mutants with their respective wild types, a ~22% reduction was observed in seedling emergence, as well as a decrease in plant density (Hulke et al., 2004). Anderson and Fehr demonstrated that the growth environment strongly influences the performance of low phytic acid cultivars: data collected in a tropical environment (Puerto Rico) were statistically different from those taken in a temperate environment (Iowa), where germination and seedling emergence were higher (Anderson and Fehr, 2008). With the aim of overcoming the reduced seedling emergence, Spear and Fehr proposed backcrossing as a strategy to obtain *lpa* progeny with unchanged seedling emergence (Spear and Fehr, 2007). Moreover, they highlighted a greater susceptibility to seedborne fungal infections in *lpa* lines during germination, which could lead to reduced field emergence (Spear and Fehr, 2007).

### Common Bean

Among the species analyzed so far, common bean was the first characterized by mutations in PA-MRP transporters that did not seem to cause negative pleiotropic effects. Over the years, two mutants have been isolated by chemical mutagenesis: *lpa1* (also known as *lpa280-10*) (Campion et al., 2009) and *lpa1^2^*, initially identified as 08IS-1281 mutant line (Cominelli et al., 2018). The *lpa1* mutation is caused by the defective *PvMRP1* gene and is characterized by a missense mutation in TMD2, that leads to E1155K amino acid change. In the allelic mutant *lpa1^2^*, a single base change occurs in TMD1 resulting in a non-sense mutation, and consequently in a truncated protein. In both of these two mutants, PA reduction (90% in *lpa1* and 75% in *lpa1^2^*) is followed by a proportional increase in free P_i_, while the total P remains unchanged. Despite this drop in PA content, the agronomic performance of the *lpa1* mutant was found to be the same as that of the wild type, or even better (Campion et al., 2009; Campion et al., 2013). This seems to be due to the presence of the *PvMRP2* paralogue, which would complement the absence of a functional *PvMRP1* gene in all plant organs except in the seed (Panzeri et al., 2011; Cominelli et al., 2018). *PvMRP2* is a highly conserved orthologous gene of *Gm13g18960*, and the proteins they encode share more than 80% of similarity with *PvMRP1*, while the similarity shared with *AtMRP5* and *ZmMRP4* proteins is lower (Panzeri et al., 2011).

In the agronomic trials carried out by Campion and co-workers, no significant differences were found in the agronomic parameters measured on the seed and on the plant (Campion et al., 2009; Campion et al., 2013). Germination tests carried out under ageing (45°C and 100% relative humidity for 48 and 96 h) and stressing environmental conditions (0.4 M NaCl treatment) demonstrated that *lpa1* does not show significant differences compared to the wild type. In particular, a lower MGT (mean germination time in hours) value in the mutant pointed out that there was even a germination response which was faster than in the parental genotypes (Campion et al., 2009). In essence, in the different growth environments tested (growth chamber, greenhouse and open field), this common bean *lpa1* mutant was not shown to be associated with any negative pleiotropic effects and to be able to afford the same good results as the wild type as concerns seedling emergence, plant growth, and grain yield. It was also shown that this mutant is hypersensitive to abscisic acid at germination (Panzeri et al., 2011).

Moreover, the common bean *lpa1* mutant has a higher drought resistance index (Chiozzotto et al., 2018). In Arabidopsis and common bean, mutations in *AtMRP5* and *PvMRP1* genes respectively, confer increased tolerance to drought. Interestingly, stomata of the Arabidopsis *mrp5-1* mutant leaves showed reduced sensitivity to light compared to the wild type ones, with the consequence of closer stomata under standard growth conditions (Klein et al., 2003). At a macroscopic level, the guard cell phenotype of the *mrp5-1* mutant confers reduced water loss from detached rosette leaves, reduced transpiration rate, improved water use efficiency, and enhanced drought stress tolerance (Klein et al., 2003). Electrophysiological measurements demonstrated that the Arabidopsis mutation impairs both ABA and cytosolic Ca^2+^ activation of slow (S-type) anion channels and ABA activation of Ca^2+^ permeable channel currents in the plasma membrane of guard cells (Suh et al., 2007), suggesting that *AtMRP5* is a central regulator of ion channels of ABA and Ca^2+^ signal transduction in guard cells. In a model proposed by Nagy et al. (2009), PA would induce the release of Ca^2+^ from the vacuole to the cytosol and would block K^+^ flux from inward channels. *AtMRP5* is necessary to transport PA from the cytosol to the vacuole, thus avoiding the continuous PA signaling. Mutations in *AtMRP5* would affect PA export into the vacuole, causing an increase of PA concentration in the cytosol. Cytosolic PA might bind to Ca^2+^ and other divalent cations and/or may induce a continuous Ca^2+^ release, thus disturbing the Ca^2+^-dependent signaling pathway. Moreover, it may reduce K^+^ uptake into guard cells by inhibiting K^+^ inward rectifying channels. It is not clear why in Arabidopsis and common bean, mutations in *AtMRP5* and *PvMRP1* genes respectively, confer increased tolerance to drought, (although most likely through different mechanisms), while for the mutation in the maize *ZmMRP4* gene the opposite was shown, and further studies are required to understand the reason for this discrepancy. However, a clarification of these aspects may help in defining strategies to develop crop *lpa* mutants.

The above cited positive results reached in common bean prompted researchers to investigate the nutritional potential of *lpa1* through *in vitro* and *in vivo* trials, aimed at verifying whether it may improve the bioavailability of micronutrients, particularly iron. As regards *in vitro* trials, in 2013 Campion and co-workers introduced the *lpa1* trait in common bean lines harbouring the Lf (lectin free) trait and producing white or colored (brown or black) beans. Then they used the Caco-2 (human epithelial colorectal adenocarcinoma cells) model to measure the amount of iron adsorbed by these cultured cells from administered bean extracts. Results showed that the bioavailable iron in *Lf + lpa* white bean seeds is on average twelve times higher than in wild type as well as in *Lf +* *lpa* colored seeds. These results, although “much an *in vitro* test is worth”, seemed to have disclosed a promising key tool to improve iron bioavailability from common bean. Indeed, a prompt confirmation arrived when Petry et al. (2016) published a paper describing an *in vivo* trial carried out on young, non-iron deficient women fed with a porridge made with wild type or *lpa 280-10* beans and cooked in boiling water for almost 2 h. Iron absorption, measured as erythrocyte incorporation of stable iron isotopes (Fe^57^, Fe^58^) from the *lpa* line, was found to be 50% higher and the total amount of iron absorbed per test meal was 85% higher than from wild type beans (Petry et al., 2016).

Despite the good agronomic performances of the common bean *lpa* mutants, undesired pleiotropic effects were described regarding their use in human diets. A second study carried on by the same group among Rwandese women proved that, while supplying diet with *lpa* beans is beneficial to iron absorption (as it happens if biofortified beans with increased iron content are used), *lpa* beans also cause adverse gastrointestinal symptoms, due to a hard-to-cook (HTC) phenotype, likely caused by the thermal stability of lectins in these lines (Petry et al., 2016). A recent publication further investigates the origin of the HTC phenotype in *lpa1* lines (Cominelli et al., 2020a). The observed HTC phenotype in *lpa1* was shown to be correlated with the redistribution of calcium cations within the seed, providing evidence for the “phytase-phytate-pectin” hypothesis; according to this idea, the reduction of PA chelating activity (due to increased phytate dephosphorylation or to reduced phytate content) determines a migration of divalent cations to cell-wall-middle lamella, resulting in the formation of insoluble pectate complexes that harden the cell walls. The authors confirmed how *lpa1* mutation also reinforces the thermal stability of seed lectins, in particular homotetramers of the antinutritional phytohemmaglutinin L (PHA-L), but not homotetramers of phytohemmaglutinin E (PHA-E) or heterotetramers made up by PHA-L and PHA-E.

Regarding the *lpa1^2^* mutant isolated in 2018, preliminary experiments conducted under controlled conditions would suggest that the effects of the mutation are similar to the ones already described for *lpa1* (Cominelli et al., 2018), while no investigation has so far been carried out to verify its nutritional features.

## Future Perspective and Conclusions

It is by now established that there are three main classes of negative pleiotropic effects caused by MRP *lpa* mutations: those affecting seed viability and lowering grain yield in cereals such as maize, rice and wheat, those related to seed emergence in soybean and those affecting important post-harvest qualities of common bean, such as cooking properties and lectin harmlessness.

Which are the strategies so far adopted and currently underway to try to eliminate or at least decrease substantially these effects?

As pointed out above, maize and rice MRP *lpa* mutants are characterized by low seed viability and reduced seedling emergence. As discussed in the previous sections, these defects may be partially or wholly attributable to an anomalous quantity of free iron cations in the seeds of the mutants and to the consequent high level of toxic ROS originated following the Fenton reaction. A possible and obvious approach to defend any human or plant cell from ROS consists in scavenging these toxic free radicals by means of molecules endowed with antioxidant properties. Thus, aiming at improving the agronomic performance of these mutants it might be sufficient to use classical breeding methods to introgress the ability to synthesize and accumulate natural antioxidants, such as carotenoids or polyphenols in the living tissues of the grain.

In small grain cereals (e.g. wheat, barley, oat, and rye) the problem of the “low yield” associated with the MRP *lpa* genotypes could be simply solved by specific breeding programs. In fact, almost all the work conducted on field performance of *lpa* genotypes present limited data using inbred lines without or with limited breeding activity. Furthermore, these data are often collected in greenhouses/growth chambers or in small experimental fields and frequently with few replications over the years. Last but not least, comparisons are in almost all cases between inbred lines in different genetic backgrounds, where many genetic differences impacting agronomic performance can take place. One of the best ways to compare *lpa* trait and yield (or any other trait, such as nutritional quality) is the comparison between sibling near-isogenic lines (homozygous wild type and *lpa*) obtained by backcrossing and selecting for yield. Nevertheless, several research programs are in progress with the aim to develop *lpa* varieties by conventional breeding and transgenic/genome editing methods and certainly new *lpa* varieties will be released soon.

As concerns soybean, Spear and Fehr (2007) suggested that backcrossing may represent an effective strategy aiming at the development of low-phytate lines devoid of negative pleiotropic effects derived from the donor *CX1834*. In a recent paper (Boehm et al., 2017), the agronomic performance of two low-phytate lines (*56CX-1273* and *56CX-1283*) obtained through five backcrosses was compared to two high-yielding elite cultivars. As expected, the agronomic trials performed in six locations highlighted that there were no significant differences in field emergence and seed yield.

For common bean, the problems of the MRP *lpa1* trait are not linked to the viability of the seeds or agronomic performances of the plants, but instead to the increase in cooking time and to the risk of lectin poisoning upon multiple consumption of meals prepared with *lpa1* beans (Petry et al., 2016). The former phenotype is caused by the hardening of the cell walls due to redistribution of Ca^2+^ ions, while the latter is linked to the higher thermal stability shown by the PHA-L lectin (Cominelli et al., 2020a). Classical breeding approaches are expected to work properly to avoid these problems. In fact, the HTC trait is strongly genotype dependent (Cichy et al., 2019) therefore introducing the *lpa1* trait in a genotype without HTC defect should not affect too much the cooking times, as shown by Cominelli et al. (2020a). As to the second problem, it may easily be solved by taking into account the importance of avoiding PHA-L containing genotypes when introgressing the *lpa1* trait in breeding programs.

Furthermore, the *lpa1* mutant root system has been little taken into consideration. Attention was mainly focused on the aboveground parts of the plant, while the underground part was neglected. However, a greater susceptibility of the mutant plants to water stress has been reported (Cerino Badone et al., 2012). Genetic analyses conducted on maize revealed that some genes (*rtcs*, *rtcl*, *rum1*), involved in auxin signal transduction, are fundamental elements for the development of lateral, seminal, and shoot-borne roots (Hochholdinger et al., 2018). Future research should focus more deeply on these genes, modulating their expression in *lpa1* mutants with traditional breeding approaches, or with their specific modulation through genome editing and GMO techniques.

In some cases, an approach different from the classical breeding was used, and *MRP* gene activity was manipulated by transgenic techniques. In particular, seed-specific silencing was conducted on *ZmMRP4* and *OsMRP5* (Shi et al., 2007; Li et al., 2014). In maize, the embryo-specific promoters *Ole16* and *Glb* were used to generate transgenic lines. *Ole::MRP4* constructs were shown to be associated with a 68–87% reduction in PA, while this decrease ranged from 32 to 75% in *Glb::MRP4* transformants. Gene-silencing constructs under the control of *Glb* tended to germinate normally and no significant reduction in seed weight was recorded (Shi et al., 2007). However, no positive agronomic results were obtained in rice with a transgenic approach such as silencing of *OsMRP5* (Li et al., 2014). The *Ole18* promoter, active both in the embryo and in the aleurone, was used for *OsMRP5* silencing and a PA reduction (35.8–71.9%) was observed in the transgenic lines. This decrease was accompanied by a strong increase (up to 7.5 times) in P_i_. Comparing these transgenic plants to the respective null siblings, a decrease in seed weight accompanied by reductions in seed germination and seedling emergence was reported (Li et al., 2014). Hence, this strategy does not appear to be effective in rice, unlike that previously reported in maize (Shi et al., 2007), probably due to the different promoters used in the two cereals. In fact, *Ole18* is active not only in the embryo, but also in the aleurone and in the endosperm (Li et al., 2014).

The main conclusion emerging from the above survey of literature is that, with the partial exception for the significant results reported in the case of common bean (Campion et al., 2009; Campion et al., 2013), the goal of achieving MRP *lpa* mutants endowed with no negative pleiotropic effects on a good field performance has not yet been reached for many crops. Moreover, this review highlights that the pleiotropic effects linked to the MRP *lpa* trait not only concern the physiology of seeds and plants, but also affect other aspects connected with the cooking properties and the harmlessness of the grain for consumers.

In conclusion, further breeding work will be necessary to attenuate the negative pleiotropic effects impacting on plant and seed performance before the development of a commercial variety that, to our knowledge, is only near to be released for common bean.

## Author Contributions

RP proposed and designed the review. FC designed the review, prepared the figures, and wrote the manuscript. DP, EC FS, and EN wrote the manuscript. All authors contributed to the article and approved the submitted version.

## Funding

This work was partially supported by MIND FoodS Hub to RP and by sPATIALS^3^ to FS and EC, both projects funded by the European Regional Development Fund under the ROP of the Lombardy Region ERDF 2014–2020—Axis I “Strengthen technological research, development and innovation”—Action 1.b.1.3 “Support for co-operative R&D activities to develop new sustainable technologies, products and services”—Call Hub; CNR-DISBA project NutrAge (project nr. 7022) to FS and EC and EU-H2020 “CropBooster-P” (grant agreement 817690) to FS.

## Conflict of Interest

The authors declare that the research was conducted in the absence of any commercial or financial relationships that could be construed as a potential conflict of interest.
