# Supplementary material for: MRP Transporters and Low Phytic Acid Mutants in Major Crops: Main Pleiotropic Effects and Future Perspectives
Source: Front Plant Sci. 2020 Aug 27;11:1301. doi: 10.3389/fpls.2020.01301 (PMC7481554; doi:10.3389/fpls.2020.01301)
Supplement: Supplementary file 1 [file Table_1.docx]

| Sup. Mat. Table 1. Main features of MRP-type ABC transporters involved in PA transport. | | | | | | |
| --- | --- | --- | --- | --- | --- | --- |
| **Species** | **Gene** | **Origin of the mutation** | **Mutant** | **PA reduction** | **Pleiotropic effects** | **Reference** |
| *Zea mays* | *ZmMRP4* | EMS | *lpa1-1*  *lpa1-241*  *lpa1-7* | 66%  >80%  >80% | Seed weight and density reduction, alteration in roots.  Reduced germination and seed density, susceptibility to oxidative stress, leaves alteration and defective primary root. | Raboy *et al.,* 2000;  Cerino Badone *et al.,* 2012  Landoni *et al.,* 2013  Pilu *et al.,* 2005;  Doria *et al.,* 2009;  Cerino Badone *et al.,* 2012;  Landoni *et al.,* 2013 |
| *Oryza sativa* | *OsMRP5* | γ rays + sodium azide  T-DNA insertion | *lpa2-1*  *lpa2-2*  4A-02500 | 20%  >90%  90% | Reduced vigor, grain weight and field emergence.  Lethal  Lethal | Zhao *et al.,* 2008  Xu *et al.,* 2009  Xu *et al.,* 2009 |
| *Triticum aestivum* | *TaABCC13* | Constitutive RNAi | *TaABCC13* | 22-34% | Delayed germination, reduced kernel viability, decreased grain filling and early emergence of lateral roots. | Bhati *et al.,* 2016 |
| *Glycine max* | *GmMRP3*  *GmMRP19*  *GmMRP13* | EMS  No reported mutant | CX1834  No reported mutant | 80% | Reduced seedling emergence and decreased plant density. Greater susceptibility to fungi infections. | Hulke *et al.,* 2004;  Spear and Fehr, 2007  Panzeri *et al.,* 2011 |
| *Phaseolus vulgaris* | *PvMRP1*  *PvMRP2* | EMS | *lpa1*  *lpa1^2^* | 90%  75% | No differences in seedling emergence and grain yield and no effect under stress condition. Faster germination response and higher drought resistance index  Preliminary experiments suggest similar effects to *lpa1* | Campion *et al.,* 2009;  Panzeri *et al.,* 2011;  Petry *et al.,* 2016; Chiozzotto *et al.,* 2018;  Cominelli *et al.,* 2020  Cominelli *et al.,* 2018 |
